# Effectiveness of Copper Nanoparticles in Wound Healing Process Using In Vivo and In Vitro Studies: A Systematic Review

**DOI:** 10.3390/pharmaceutics14091838

**Published:** 2022-08-31

**Authors:** Cristian Sandoval, Gemima Ríos, Natalia Sepúlveda, Jessica Salvo, Vanessa Souza-Mello, Jorge Farías

**Affiliations:** 1 Escuela de Tecnología Médica, Facultad de Salud, Universidad Santo Tomás, Los Carreras 753, Osorno 5310431, Chile; 2 Departamento de Ingeniería Química, Facultad de Ingeniería y Ciencias, Universidad de La Frontera, Temuco 4811230, Chile; 3 Departamento de Ciencias Preclínicas, Facultad de Medicina, Universidad de La Frontera, Temuco 4811230, Chile; 4 Carrera de Enfermería, Facultad de Ciencias de la Salud, Universidad Autónoma de Chile, Temuco 4811230, Chile; 5 Programa de Doctorado en Ciencias Morfológicas, Facultad de Medicina, Universidad de La Frontera, Temuco 4811230, Chile; 6 Laboratorio de Morfometría, Metabolismo y Enfermedades Cardiovasculares, Centro Biomédico, Instituto de Biología, Universidade do Estado do Rio de Janeiro, Rio de Janeiro 22775-000, Brazil

**Keywords:** angiogenesis, antimicrobial, regeneration, nanoparticles

## Abstract

Chronic wounds are defined as wounds that do not heal in an orderly and timely manner through the various stages of the healing process. Copper nanoparticles are essential in dressings for wound healing because they promote angiogenesis and skin regeneration, which hasten the healing process. This systematic investigation sought to explain how copper nanoparticles affect chronic wound healing in vivo and in vitro. We realized a systematic review of original articles studying the effectiveness of copper nanoparticles in the healing process of chronic wounds. The protocol was registered in the PROSPERO database. Several databases were searched between 2012 and January 2022 for English-language papers using MeSH terms and text related to chronic wounds, copper nanoparticles, and wound healing. Quality was evaluated using National Institute for Health and Care Excellence methodology and PRISMA guidelines. We looked at a total of 12 primary studies. Quantitative data were gathered and presented in all studies. Our results suggest that copper nanoparticles could have an excellent healing property, facilitating the liberation of growth factors that help the anti-inflammatory process of the wound and significantly improving antibacterial and antioxidant activities. In addition, copper presents a higher biocompatibility than other metallic ions, promoting regeneration and increasing skin quality.

## 1. Introduction

Chronic wounds are characterized as either failing to move through a well-ordered and suitable reparative process to produce anatomic and functional integrity within three months or continuing through the repair process without creating a sustained anatomical and functional outcome [1,2]. Chronic wounds are divided into four groups based on the etiologies that cause them: venous, arterial insufficiency, pressure, and diabetic ulcers [3]. Due to their rising prevalence and high management cost, chronic wounds are significant to the healthcare system. According to a study evaluating the cost, effect, and Medicare policy implications of chronic non-healing wounds, 8.2 million Medicare members in the United States are afflicted [4]. In recent years, impregnated dressings have enhanced wound care by enabling wound closure through in vivo and in vitro studies [5,6,7,8,9]. Moreover, there has been increased interest in using copper (Cu), gold, and silver metal nanoparticles (NPs) to limit the spread of infectious processes by impeding protein synthesis, oxidizing cell membranes, and damaging the nucleic acids of bacteria and viruses [10,11].

Gold nanoparticles are biocompatible and are now employed in medication delivery, photothermal treatment, and tissue regeneration. However, gold nanoparticles (AuNPs) containing polycaprolactone nanofibers have a limited antibacterial action against *Staphylococcus aureus*, *Escherichia coli*, *Pseudomonas aeruginosa*, and *Candida albicans* (zone of inhibition values less than 3 mm) [12]. Furthermore, while AuNPs have the ability to limit bacterial growth and promote wound healing, they should be utilized in conjunction with other antibacterial chemicals or particles [13,14].

Silver nanoparticles can be used in a variety of goods, including bandages, gauzes, sutures, plasters, and many more lotions and ointments for wound healing [15]. Along with antibacterial capabilities, silver-treated textile materials and surgical sutures display better wound healing properties in vitro, demonstrating that silver has a favorable influence on cell migration and proliferation [16,17,18]. However, silver nanoparticles are prone to aggregation, which may result in a change in size and a loss of antibacterial activity. As a result, the creation of materials with evenly dispersed silver in the polymer matrix is a hot area for future study [19].

Several metabolic activities call for modest amounts of Cu, an essential element [10,20]. In fact, under controlled circumstances, Cu performs a significant function in the healing activity by stimulating the expression of molecules found in the extracellular matrix, including integrins, the primary moderators of cell connection to the extracellular matrix, fibrinogen, and collagen formation [10,20,21,22]. However, using Cu excessively might be harmful since it produces free radicals that can produce cell death and lipid peroxidation [23,24]. For instance, just 3% of breast epithelial cells cultivated with 10 mg of Cu in nanofibers survived, indicating that the quantities of Cu released from the nanofibers are highly hazardous to cells in tissue culture [25].

Silver is a metal poorly digested by the human body; however, Cu possesses strong biocidal characteristics [26]. More significantly, Cu is essential for skin regeneration and angiogenesis [27,28]. In animal models, Cu hastens the healing process by promoting angiogenesis and vascular endothelial growth factor (VEGF) [29] through hypoxia-is-induced factor-1-alpha (HIF-1) action, where Cu increases HIF-1 expression [10] and HIF-1 binding to the essential elements in the putative enhancer and promoter regions of genes controlled by HIF-1 [30].

The two main protease groups involved in wound healing are serine proteases and matrix metalloproteinases (MMPs) [31,32]. High Cu concentrations have been observed to promote MMPs expression in fibroblasts, but low Cu levels have been reported to increase MMPs activity [33]. According to certain studies, Cu can up-regulate MMP2 and MMP3, even in a large amount of metal that suppresses MMP activity [33,34]. In essence, Cu ions might promote VEGF expression and angiogenesis, which would aid in wound healing [35]. Accordingly, copper sulfide (CuS) NPs may also be capable of photothermal therapy, which helps eradicate bacteria with a non-resistant and minimally invasive approach by near-infrared (NIR) light irradiation action [36,37]. Therefore, we aimed to assess the effectiveness of Cu NPs in the healing process of chronic wounds using in vivo and in vitro models during the last ten years.

## 2. Material and Methods

A systematic review of quantitative research studying the role of Cu NPs in wound healing for chronic wounds using in vivo and in vitro studies. The protocol was recorded in PROSPERO, CRD42022341892. The review is described by PRISMA [38].

### 2.1. Search Strategy and Selection Criteria

#### 2.1.1. Search Strategy

Using MeSH terms (“wound healing” AND “copper nanoparticles” AND “chronic wounds”) and text words relating to the role of Cu NPs in wound healing for chronic wounds by the research question, several databases (MEDLINE, EMBASE, Scopus, and Web of Science) were examined from January 2012 to January 2022 for original articles in English. The reviews covered a variety of biological activities, including antifungal efficacy and antimicrobial, antioxidant, anti-inflammation, and wound healing investigations. Additionally, the reference lists of included studies and relevant reviews were searched.

#### 2.1.2. Identification of Relevant Studies

Two reviewers evaluated titles, abstracts, and papers for inclusion or exclusion. Discussion with a different reviewer helped to settle any discrepancies between the results of the reviewers.

#### 2.1.3. Types of Study and Design

The studies must have reported the role of Cu NPs in wound healing for chronic wounds or the effects of Cu NPs in biological activities and must have been English studies. The inclusion criteria were: (1). primary studies with a quantitative component; (2). studies using descriptive or inferential statistics approaches, with parametric or non-parametric methods; and (3). clinical trials, experimental studies, cross-sectional studies, or randomized controlled trials. Studies were excluded if they met the following criteria: (1). did not include or specify numerical data; (2). were not original investigations published in full; (3). were not published in a peer-reviewed journal; (4). conference abstracts; (5). systematic reviews; (6). editor letters; and (7). studies that did not focus on the role of Cu NPs in wound healing for chronic wounds, or those that did not describe antifungal efficacy and were not antimicrobial, antioxidant, anti-inflammation, and wound healing studies.

#### 2.1.4. Population

For in vivo studies, animal models included only healthy participants, evaluating Cu NPs and their protective capacity on wound healing for chronic wounds or the effects of Cu NPs on biological activities. Pregnant animals, those with burns in other places than the skin, and/or those with comorbidity were excluded.

For studies on the human population, studies that focused just on postmenopausal women or unhealthy participants were excluded.

For in vitro studies: HDFs, HEKas, HEK, HUVECs, recombinant human bFGF, NIH-313, HFF-1, HaCaT, HCEPC, NIH-313, HEK293, bacterial cellulose (BC), and L929 cells exposed to Cu NPs were included, evaluating their protective capacity on wound healing for chronic wounds or the effects of Cu NPs in the biological activities.

#### 2.1.5. Quality Assessment/Risk of Bias

One reviewer (GR) assessed quality using the National Institute for Health and Care Excellence (NICE) methodology [39] and another reviewer (NS) analyzed it for accuracy. Disparities between authors were solved by discussion. No studies were excluded based on assessment.

#### 2.1.6. Data Extraction and Synthesis

Data relating to the population and study characteristics of the included studies were extracted by one reviewer and checked by another reviewer (Table 1).

Two researchers went line by line through the results and discussion sections of each text to look for data involving the role of Cu NPs in the healing process or the effects of Cu NPs in biological activities to find information relevant to variables involved in the role of Cu NPs in the healing process using in vivo and in vitro studies. The text was reviewed in greater detail and rearranged into topics (Table 2). These were included if the study’s authors built their interpretation and concepts from the initial data.

## 3. Results

**Figure 1:**
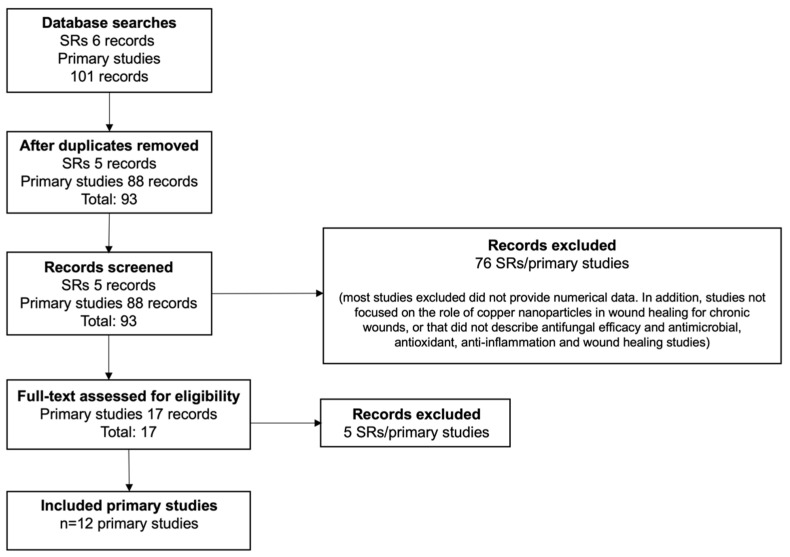
PRISMA flow diagram. SR: systematic review.

The flow chart of the selection process of the studies is shown in Figure 1 [40,41,42,43,44,45,46,48,49,50,52,53]. Table 1 lists the studies that were included, along with the demographics, environments, and contexts in which they were carried out.

### 3.1. Description of Included Studies

Eight publications from the original studies were carried out in China, two in the US, one in Canada, and one in India.

A total of 12 original articles were analyzed. The papers collected and reported quantitative data through clinical trials or experimental studies (Table 1). Ten studies have used in vivo and in vitro models [41,43,44,45,46,48,49,50,52,53] while two have used in vitro studies [40,42]. Details of chronic wounds of each study, where available, are shown in Table 1.

The above-mentioned in vitro studies used human umbilical vein endotelial cells [43,44,49,52,53], immortalized human epithelial keratinocytes [48,50,52], human dermal fibroblasts [50,52], diabetic (db/db) mice and non-diabetic mice (C57BL/6) [50,52], human embryonic kidney [41], human keratinocytes cells [44], NIH-3T3 cell line [45,49], bacterial cellulose [40], human dermal fibroblasts extracted from split-thickness skin biopsies and obtained during breast reduction and abdominoplasty [42], human foreskin fibroblast cells [43], human corneal epithelial cells [44], fibroblast cell line [46], and recombinant human basic fibroblast growth factor [49]. For the in vivo models, female BALB/c mice [41,43,44,53], type 1 diabetic mice [48], Sprague-Dawley rats [45,49], and Wistar rats [46] were used.

### 3.2. Quality Assessment

Table 3 displays the quality assessment outcomes and evaluation standards for the studies. The studies’ overall quality for internal and external validity was often high or moderate. No studies were disqualified due to poor quality.

### 3.3. Relation between NPs and Wound Treatment

The main advantages of Cu NPs used for wound treatment are described in Table 2. Nanoparticles with antibacterial qualities and low toxicity are excellent for use in wound dressings (Table 1). Different assays have been used to evaluate Cu tolerance, Cu toxicity, in vivo biocompatibility, and in vitro cell viability [40,41,42,43,45,46,48,49,50,52]. In addition, the antibacterial response was evaluated through different methods such as brain heart infusion [42], growth-inhibition assay [43], transmission electron microscopy [43], SYTO9/propidium iodide live/dead fluorescent staining assay [44], Luria-Bertani broth [45], or the disc diffusion method [40,46]. Finally, wound healing has been analyzed using the aortic ring assay [42], chick chorioallantoic membrane assay [42], optical coherence tomography angiography [50], photoacoustic imaging in vivo [48], immunohistochemistry [45,48,49], dermal excision wound model [52], matrigel assay [44], histochemical techniques [41,45], and ELISA kit [46].

### 3.4. Cytotoxicity Assays

Monitoring cell growth inhibition was assessed through cytotoxicity evaluation. However, cell cytotoxicity could be evaluated using other parameters, as shown in Table 1. In this sense, cells exposed to hollow mesoporous CuO nanospheres that resemble viruses (HvCuO@GOx) showed negligible cytotoxicity and biocompatibility when different glucose concentrations (2 mM, 4 mM, 6 mM, 8 mM, 10 mM) were used [48]. Likewise, the Cu nanodots (CuS NDs) dose did not effect the viability of the cells with or without laser irradiation. Even at a dosage of 45 g/mL, both cell lines were still alive, demonstrating the very low cytotoxicity of CuS NDs and photocytotoxicity [43].

In vitro assays have shown that the increase in human dermal fibroblasts’ (HDFs) migration due to folic acid-modified Cu-based metal–organic framework (F-HKUST-1) exposure could result from the persistent release of Cu^2+^ and folic acid along with modest cytotoxicity [52]. At the same time, according to CCK-8 analysis, a small quantity of ultrasmall Cu_5.4_O NPs (USNPs) were able to shield the cells completely from 250 μM H_2_O_2_; meanwhile, HEK293 cells had normal polygonal cytoskeleton morphology after being treated to 200 ng mL^−1^ Cu_5.4_O USNPs for 48 h, showing high biocompatibility [41]. Finally, while BC/Cu_20_ membranes did not exhibit cytotoxicity to normal human dermal fibroblasts (NHDFs), BC/Cu_60_ and BC/Cu_100_ membranes drastically reduced cell viability [40].

### 3.5. Antibacterial Response

The human body uses Cu for the innate immune response by boosting the bactericidal and phagocytic functions of neutrophils and the antimicrobial activity of macrophages [54]. In this sense, the quantity of biofilm present following treatment with Cu-containing mesoporous bioactive glasses (Cu-MBG) is reduced for both species. However, in the infected skin model, the effect of Cu-MBG on *Pseudomonas aeruginosa* was noticeably less pronounced than its effect on *Staphylococcus aureus* [42]. In the case of methicillin-resistant *Staphylococcus aureus* (MRSA) and extended-spectrum β-lactamase (ESBL) *Escherichia coli* treated with CuS NDs and NIR irradiation, where ultrasmall NDs stuck to the bacterial surface, their original form was distorted and revealed wrinkled bacterial cellular wall/membranes with visible lesions and holes, which may be due to the strong contact between CuS NDs and the bacterial cell wall [43].

Samples with higher Cu displayed bigger inhibition zones when the antibacterial activity was measured using the disk diffusion method. BC/Cu_20_, BC/Cu_60_, and BC/Cu_100_ had diameters of 14.7 mm, 18.0 mm, and 21.3 mm, respectively [52]. In comparison to hydrogel composite, the gelatin + CuO hydrogel (3.8 ± 0.3 in *Staphylococcus aureus* and 4.8 ± 0.7 in *Escherichia coli*) and gelatin + ZnO hydrogel (4.9 ± 0.6 in *Staphylococcus aureus* and 5.3 ± 0.2 in *Escherichia coli*) showed greater zones of inhibition [46]. Likewise, the viabilities of *Staphylococcus aureus* and *Escherichia coli* were reduced when the concentrations of Cu nanoparticles were added to hydrogel samples (93.7% to 9.2% and 92.4% to 8.8%, respectively) [45]. Finally, F-HKUST-1 slowly released Cu^2+^ during wound healing processes, which is recognized for having an impact on the expression and synthesis of growth factors, matrix metalloproteinases, collagen, elastin, and integrins [52].

### 3.6. Wound Healing

Wound evaluation has historically relied on a visual inspection by the trained clinician. However, new elements provide accurate assessment modalities. In this sense, the average small vessel length increased significantly for both Cu-MBG and mesoporous bioactive glasses (MBG) [42]. As evidence of HvCuO@GOx (HSHvCuO@GOx) superiority in new vessel development, CD34-positive cells in the HvCuO@GOx group’s adhesive hydrogel were higher than those in the pure hydrogel and HSHvCuO groups [48].

On days 7, 14, and 28, it was shown that the composite scaffold’s rate of wound contraction (13.2 ± 1.4 mm, 8.5 ± 2.9 mm, and 0.0 ± 0.01 mm, respectively) was significantly higher than cotton gauze’s (17.7 ± 3.2, 12.3 ± 2.6, and 3 ± 4.7 mm, respectively) [46]. Similarly, the wound treated with laser and AuAgCu_2_O NSs shrank significantly, indicating a 93.5% healing rate [44]. In fact, an almost completely healed wound in a diabetic mouse model using HSHvCuO@GOx gauze has been found. However, wounds treated with control and HvCuO-based hydrogel did not recover, showing that the HvCuO@GOx-based hydrogel can successfully promote the healing of *Staphylococcus aureus*-infected wounds [48]. Finally, the incorporation of folic acid into HKUST-1 also reduces Cu^2+^ toxicity in vivo, as has been described [52].

## 4. Discussion

This systematic review compiles and synthesizes the evidence of twelve quantitative studies that relate the activity of Cu NPs with their different types of antibacterial, cytotoxic, and proangiogenic properties in in vivo and in vitro studies for chronic wound healing.

### 4.1. Summary of Key Findings and Interpretation

The four overlapping healing processes during wound healing are hemostasis, inflammation, proliferation, and remodeling [55]. Both internal and environmental causes may hamper the healing process. Significant factors that prevent wound healing include microbial infections and reactive oxygen species (ROS). They might prolong each recovery step, resulting in less than ideal structural and functional outcomes [56].

In this regard, it is crucial to note that chronic, non-healing wounds have been associated with impaired healing when higher and continued ROS have been detected in vivo [57]. On a molecular level, high ROS and reactive nitrogen species (RNS) can affect the activity of dermal fibroblasts and keratinocytes as well as change and/or degrade extracellular matrix (ECM) proteins both directly and indirectly (through activation of proteolysis). This is in addition to ROS-mediated transcription, which can cause matrix metalloproteases to be induced and prolong pro-inflammatory cytokine secretion [58]. In fact, the adequate equilibrium between low and high levels of ROS is essential in defining functional outcomes: although low levels of ROS are required for promoting efficient healing [59], excessive ROS release damages cells and hinders wound repair [60]. Instead of directly affecting ROS, one option for wound healing might involve influencing the antioxidant system. The in vitro studies analyzed did not exhibit noticeable cytotoxicity during low levels of Cu NPs treatment because it was well-tolerated [40,41,42,43,45,46,48,49,50]; it does not deform the structure of the cell membrane, and it can be used to relieve oxidative stress at the wound site. However, this is only achieved if a Cu ion is administered in controlled release [50]. In fact, if similar amounts are administered in a short period of time and abruptly, the Cu NPs can become toxic to cells and cause apoptosis [50].

Copper-based combined pharmacological complexes are more effective as antibacterial, antifungal, and antiviral medicines [61,62]. Copper concentration has a direct relationship with the mechanism by which it has a bacteriostatic or bactericidal effect [63]. The highest recorded impact was for copper metal (99.9%), and these results were seen in alloys having at least 70% copper [61,62]. However, due to the structure of the root canal system, which contains microecological niches such as dentinal tubules where antimicrobial drugs cannot reach, nanotechnology presents as an alternative to improve treatment success and endodontic retreatment rates [64]. Although systemic antibiotic administration helps the body fight microbial infections, a locally applied antimicrobial treatment is preferred for a wound [65]. Pomades, gels, and ointments can eliminate the germs that increased in large numbers where the damage was, potentially cutting the length of the healing process [66]. These materials are most helpful to patients with compromised immune systems, such as those with diabetes, hepatitis, and acquired immune deficiency syndrome [67,68]. In fact, according to our systematic analysis, the nanocomposite hydrogels under investigation have solid antioxidant potential against MRSA and ESBL *E. coli* as well as broad-spectrum antibacterial activity [40,43,44,45,46,49,53], including *P. aeruginosa* [42]. When a wound is in the inflammatory phase, large amounts of ROS such as superoxide (O2), peroxynitrite (ONOO−), and hydroxyl radicals (OH−) are generated that damage the body’s proteins and DNAs [69]. However, free radicals can be removed from the wound site thanks to the decisive antioxidant action. In this regard, additional research has demonstrated the ability of CuO hydrogels to eradicate human-pathogenic species of Gram-positive and Gram-negative bacteria [6,70,71]. These studies show that using these hydrogels as dressings at the location of a wound speeds up the healing process. These attributes synergistically support wound healing.

In wound therapy, primarily two types of NPs are used: (1) NPs with intrinsic properties that promote wound closure; and (2) NPs used as delivery vectors for therapeutic drugs. The former can be separated into nonmetallic nanomaterials and metallic or metal oxide nanoparticles. To repair the damaged cells and restore epidermal integrity, wound healing requires the migration and proliferation of different cells, angiogenesis, and collagen deposition processes [72]. In fact, collagen formation is a very vital step [73]. Recent investigations have unmistakably demonstrated that NPs represent a crucial therapy platform for skin wounds [41,44,45,46,50,74,75]. Cu is a well-known NP with a lengthy history of direct angiogenesis involvement and antibacterial action. Additionally, Cu has been found to have a possible involvement in the healing of wounds by controlling the expression of 84 genes linked to angiogenesis and wound repair [5]. In addition, to the best knowledge, studies have reported the effects of CuNPs on keratinocyte and fibroblast cell proliferation and migration during wound healing [41,42,48,50,52].

Increasing the characteristics of polymer nanocomposites, however, is a constant challenge for their broad usage in research [76]. Wound healing, gene therapy, tissue engineering, and controlled drug administration are only a few of the uses for such biomaterials [77]. The biodegradability, nontoxicity, biocompatibility, and environmental susceptibility of polymer nanocomposites have sparked enormous interest and advancement [78].

Because of their bioavailability and repeatability, polysaccharides (e.g., sodium alginate) are often utilized in drug delivery activities. They are biocompatible and biodegradable, with low immunogenicity, and they are attractive candidates for medicine administration [79]. In this sense, sodium alginate/polyvinyl acetate nanocomposites [80], drug-loaded nanofibers [81], and polyvinyl acetate/gelatin biopolymeric films [73] could be promising therapeutic options for preventing both resistant infections and life-threatening complications in exudative wounds.

### 4.2. Scope and Limitations

It is essential to highlight that our objective was to evaluate the use of Cu NPs in the healing of chronic wounds. However, it is necessary to consider that multiple physiological processes are involved in the healing process. Within these mechanisms are VEGF-induction, angiogenesis, and the expression and normalization of skin proteins such as collagen and keratin. As a result, the sustained release of non-cytotoxic amounts of Cu ions promotes in vivo wound healing by inducing angiogenesis, collagen deposition, and wound re-epithelialization.

Our findings confirm that Cu NP treatment promotes the formation of new vessels and significantly increases their total length, resulting in a denser and more stable vascular network at the wound site. Furthermore, with new collagen production and epithelial cell regeneration, the time spent in the inflammatory phase was reduced, allowing for a faster transition to the late stage of angiogenesis. However, the activity of Cu NPs is restricted by their biocompatibility in certain biological activities, low toxicity, and antibacterial capabilities, which are always dependent on Cu being present in low-to-moderate concentrations so that it is not damaging to target cells.

In addition, our review had other limitations, i.e., a low quantity of articles linking Cu NPs to chronic wound and wound healing were found. Also, the intervention times were highly variable between studies, with significant differences in the number of weeks and days. Finally, some studies did not provide sufficient data to compare the results obtained before and after the intervention, and some did not even incorporate the baseline measurements for the parameters studied, which limits the extraction of information.

## 5. Conclusions

Cu NPs have a high antibacterial response capacity since they are prone to interacting with the bacteria membrane. In addition, they can penetrate the bacteria biofilm and release ions inside it, compromising its integrity, where the rigidity of the membrane is an important determinant of antibacterial efficiency.

Our results have shown that Cu NPs are structurally designed to have a rough surface to facilitate adhesion to the bacterial membrane, helping to reduce or prevent the formation of bacteria. This bactericidal task occurs within a few minutes of encountering the bacteria; therefore, it is fast-acting, efficient, and long-term since it lasts over time without losing its bactericidal activity. However, it has been shown that Cu NDs demonstrate a much higher antibacterial effect than Cu NPs using laser radiation and can almost completely lyse bacterial membranes.

## Figures and Tables

**Table 1:** Characteristics of included studies.

References	Country	Population, Setting	Inter Details	Investigated Outcomes	Study Aims		Main Results
[40]	CN	BC cells were used.	BC membranes were divided into 15 mm-diameter rounds. Purified BC membranes were extensively rinsed into CuCl_2_ solution (20, 60, or 100 mM) to create the BC/Cu composite membranes.	XRD analysis, FTIR spectra, thermal stability, Cu ion release, long-term antibacterial activity, and in vitro cytotoxicity of BC/Cu membranes.	To fabricate BC/Cu composite membrane by in situ chemical reduction method.	Purified BC membranes were first immersed overnight in CuCl_2_ aqueous solutions, resulting in Cu^2+^ adhesion to BC nanofibers. Following that, NaBH_4_ was introduced to the membranes, and the Cu_2_ anchored in the membranes was instantly reduced to Cu. After 30 min, the reaction was halted, yielding a stable dark-brown BC/Cu membrane.	In BC/Cu membranes: XRD analysis: No differences were found between XRD curves of BC/Cu and cellulose I crystal. FTIR spectra: No differences were found between BC/Cu and BC membranes. Thermal stability, Cu ion release: In BC/Cu_100_, a considerable weight reduction stage was seen. Antibacterial activity: Significant inhibitions against S. aureus and E. coli after 1, 45, and 90 days were found in all BC/Cu membranes. In vitro cytotoxicity: After being exposed to the membrane extracts from BC/Cu_60_ and BC/Cu_100_, cell number was considerably reduced.
[41]	CN	HEK293 cells and female BALB/c mice were used.	In vitro: To evaluate ROS scavenging activities and biological compatibility, HEK293 cells were used. In vivo: To evaluate biocompatibility, BALB/c mice were used and 4 μg kg^−1^ Cu_5.4_O USNPs were applied. During therapeutic efficacy assessment, BALB/c mice were used in the AKI model, and they were separated as follows: PBS, 8 mg/kg, 40 mg/kg, 160 mg/kg, and they received USNPs at doses of 2 μg/kg, respectively.	In vitro: ROS scavenging activities and biocompatibility of Cu_5.4_O USNPs. In vivo: Cu_5.4_O USNPs: biocompatibility and therapeutic efficacy.	To create ultrasmall Cu-based systems, which could serve as a model for future nanosystems employed in the treatment and prevention of disorders connected to ROS.	The Cu_5.4_O USNPs were created as follows: 10 mM CuCl_2_ powders in deionized water were dissolved. Next, they were swirled for 10 min at 80 °C. Then, 100 mM L-ascorbic acid solution was added to the CuCl_2_ solution above, and the pH of the solution was adjusted to 8.0–9.0 using NaOH solution. After the process, the bigger aggregates were removed by centrifugation, and the supernatant was dialyzed against water for two days to remove tiny molecules. Centrifugation was used to concentrate purified Cu_5.4_O USNPs.	In vitro: ROS scavenging activities of Cu_5.4_O USNPs: Doses of 150 ng/mL USNPs eliminated most free radicals. Biocompatibility of Cu_5.4_O USNPs: HEK293 cells showed normal morphology after exposure to 200 ng/mL. USNPs. In vivo: Biocompatibility of Cu_5.4_O USNPs: No differences between IL-6 and TNF-α levels of USNPs and control group (*p* > 0.05). Therapeutic efficacy of Cu_5.4_O USNPs: On days 4, 7, 9, and 15 post-surgery, the wound healing rate was always faster in the USNPs group (*p* < 0.01).
[42]	CA	Human dermal fibroblasts isolated from split-thickness biopsies received from abdominoplasties and breast reduction were used.	Human primary fibroblasts were seeded in DMEM and incubated at 37 °C and 5% CO_2_. A final concentration of either 0.1 or 1 mg/mL in 1 mL of total suspension of MBG and Cu-MBG was added to the plates.	Cytotoxicity testing, antibacterial effects against planktonic bacteria, antibacterial effects against biofilms, the antibacterial action of Cu-MBG in infected skin, and evaluation of the proangiogenic activity of Cu-MBG.	To evaluate the antibacterial action, both in biofilm models and in the infected tissue-engineered skin model, and to investigate the proangiogenic action using CAM and aortic ring assays.	In double-distilled water, cetyltrimethylammonium bromide and NH_4_OH solution were dissolved by stirring for 30 min. Next, tetraethyl orthosilicate, Ca(NO_3_)_2_·4 H_2_O, and CuCl_2_ were dissolved in this solution while stirring for 3 h. Then, the particles were separated by centrifugation at 10,000 rpm for 5 min before being rinsed once with distilled water and again with ethanol. The resulting precipitate was dried for 12 h at 70 °C. Finally, the powders were calcined for five minutes at 600 °C in the air at a heating rate of 1 °C/min. The calcined powders were referred to as Cu-MBG.	Cytotoxicity tests: A reduced cellular metabolic action was found for 1 mg/mL Cu-MBG NPs. No difference for 0.1 mg/mL Cu-MBG was found. Antibacterial effects against planktonic bacteria: Significant effects against *P. aeruginosa* and *S. aureus* for 100 μg/mL Cu-MBG were found. Antibacterial effects against biofilms: Reduced biofilms of *P. aeruginosa* and *S. aureus* were found after Cu-MBG exposure. Antibacterial action of Cu-MBG in an infected skin: No difference was found in *P. aeruginosa*-treated samples and controls. Assessment of proangiogenic activity of Cu-MBG: An increased number of junctions, rate of cell outgrowth, area of outgrowth, and total vessel length in Cu-MBG-treated CAM membranes was found.
[43]	CN	HFF-1 and HUVEC cells. BALB/c and diabetic mutant (db/db) mice were used.	In vitro: Antibacterial activity: CuS NDs + NIR were used against Escherichia coli and Staphylococcus aureus. Cell migration: HFF-1 cells were used. Cell angiogenesis: HUVECs were incubated in the presence of CuS NDs. In vivo: To develop the infection model, methicillin-resistant Staphylococcus aureus suspension was used.	In vitro: Antibacterial activity, cell migration, and cell angiogenesis. In vivo: Antibacterial activity and wound healing.	To develop CuS NDs stabilized in albumin to improve healing and antibacterial effects.	CuS NDs were created using a simple one-step hydrothermal method. During the production, BSA was utilized to manage particle size and stability.	In vitro: Antibacterial activity: CuS NDs heavily inhibited Escherichia coli and Staphylococcus aureus reproduction. Cell migration: HFF-1 exhibited the greatest migration after CuS NDs+NIR treatment. Cell angiogenesis: The cells treated with CuS NDs formed fitted junctions, mesh circles, branch nodes, and parallel cell lines. In vivo: Antibacterial activity: After CuS NDs+NIR treatment, the treated area became smaller. Wound healing: On day 12 after CuS NDs+NIR treatment, the wounds almost disappeared completely.
[44]	CN	HaCaT and HCEPC cells were used. Female BALB/c mice were used.	In vitro: The antibacterial activity of AuAgCu_2_O NSs with a laser (or without a laser) was evaluated on Escherichia coli and Staphylococcus aureus. In vivo: Mice were distributed into the following groups: control group, Ag NPs hydrogel PTT group, AuAgCu_2_O NSs hydrogel group, and AuAgCu_2_O NSs hydrogel PTT group. For the skin infection, Staphylococcus aureus suspension was used.	In vitro: Antibacterial activity, the bacterial integrity disruption, antibiofilm activity, and the behavior of AuAgCu_2_O NSs. In vivo: Wound healing and toxicity evaluation.	To develop a nanoagent to improve healing and antibacterial effects.	The hollow AuAg NSs were first synthesized using the well-used Ag NP templated galvanic replacement procedure. The hollow AuAg colloids were then added to an aqueous solution of PVP and Cu(NO_3_)_2_. After 30 min of stirring, N_2_H_4_·3H_2_O solution was immediately introduced into the solution. The olive-green hollow AuAgCu2O NSs were then centrifuged and cleaned three times before being redisposed in water for subsequent usage. To create a homogenous composite gel, sodium hyaluronate powders were progressively introduced into the hollow AuAgCu_2_O dispersion solution at a sufficient concentration while stirring.	In vitro: Antibacterial Activity: The reproduction of Escherichia coli and Staphylococcus aureus was inhibited after AuAgCu_2_O NSs with laser (or without laser). Bacterial Integrity Disruption: The death of Escherichia coli was almost complete after AuAgCu_2_O NSs treatment with laser. Antibiofilm Activity: AuAgCu_2_O plus laser showed a rare signal of biofilm activity. Behavior of AuAgCu_2_O NSs: In AuAgCu_2_O NSs treatment with laser, the highest migration rate was observed. In vivo: Wound healing: On day 8, the trauma area was smaller and an increase in the vessel number after AuAgCu_2_O NSs treatment plus laser was observed. Toxicity Evaluation: AuAgCu_2_O NSs-treated RBC did not show a broken form.
[45]	CN	NIH-313 cells and Sprague-Dawley rats were used.	In vitro: Antibacterial activity and determination of reactive oxygen species: Escherichia coli and Staphylococcus aureus were used for the antibacterial experiments. Cell viability and proliferation: NIH-3T3 cells were used to evaluate the activity of the hydrogel with or without Cu NPs cuts. qPCR analysis: The mRNA expressions of IL-1β, IL- 6, TNF-α, and IL-10 was evaluated. In vivo: Sprague-Dawley rats were used to evaluate the effects of hydrogel, hydrogel treatment plus laser, Cu NP-embedded hydrogel, and Cu NP-embedded hydrogel treatment plus laser.	In vitro: Antibacterial activity, determination of reactive oxygen species, cell viability and proliferation, and quantitative real-time PCR analysis. In vivo: Wound healing and immunofluorescence staining.	To design GelMA hydrogels combined with BACA/Cu NPs to promote wound healing and antibacterial activities against Gram-positive/negative bacteria.	Cu NPs were created in a modified version by reacting Cu ions with Na_3_C_6_H_5_O_7_. CuSO_4_·5H_2_O was then vigorously agitated in ultrapure water for 2 h. Following complete dissolution, a glass container was filled with ethylene glycol and distilled water, and the pH was adjusted to 10 using a strong NH_3_ solution. The reducing agent, Na_3_C_6_H_5_O_7_ solution, was then added to the previously described combination. After that, the vial was submerged in an oil bath at 90 °C until the blue transparent solution changed to the distinctive brownish-red hue when heated. Cu NPs were cleaned three times with ultrapure water before being stored in an ethanol solution at 20 °C.	In vitro: Antibacterial activity: Cu NP-embedded hydrogels increased Cu release after NIR laser irradiation. Determination of reactive oxygen species: No differences in lipid peroxidation were found. Cell viability and proliferation: All the hydrogel + CuNPs and hydrogel + CuNPs+NIR groups showed a slight decrease in the ability to proliferate NIH-3T3 cells. qPCR analysis: No differences in IL-1β, IL-6, TNF-α, and IL-10 expression were found. In vivo: Wound healing: The wound healing was faster in the Gel-MA/BACA-Cu NPs composite hydrogels treatment plus laser irradiation.
[46]	IN	L929 cells and Wistar rats were used.	In vitro: Cytocompatibility studies: L929 cell line was used to MTT assay according to the following treatments: gelatin, gelatin + GAGs + asiatic acid, gelatin + ZnO + CuO, and developed hydrogel composite. DNA quantification: Lysis buffer was used to cell lysate on day 1 and day 3. In vivo: Wound healing: Wistar rats were used to evaluate the efficacy of the wound dressing. Evaluation of TNF-α and MMP-2: Quantification of TNF-α and MMP-2 was realized on 7th, 14th, and 28th days after damage.	In vitro: Cytocompatibility studies and DNA quantification. In vivo: Wound healing and TNF-α and MMP-2 quantification.	To develop a hydrogel platform composed of biopolymer gelatin and glycosaminoglycans combined with asiatic acid, ZnO, and CuO NPs. To evaluate the efficacy of the wound dressings in burn wounds.	Dissolving gelatin in distilled water yielded a gelatin solution. After complete dissolution, appropriate amounts of (C_14_H_21_NO_11_)n, C_13_H_21_NO_15_S, C_30_H_48_O_5_, ZnO, and CuO NPs were added sequentially and mixed for 6 h. The solution was collected on a Petri plate after homogenization and lyophilized overnight. The hydrogels were then cross-linked using EDC coupling [47], washed with distilled water, and kept in a refrigerator at 20 °C until use.	In vitro: Cytocompatibility studies: From day 1 to day 3, an increase in cell numbers was observed in GAGs and the asiatic acid hydrogel group. However, a slow proliferation in cell number was observed in ZnO and CuO NP scaffolds. DNA quantification: From day 1 to day 3, an increased L929 cell number was found in the gelatin + GAGs + asiatic group. In vivo: Wound healing: On day 28, in the hydrogel composite group, a complete healing was observed. Evaluation of TNF-α and MMP-2: On day 7, a lower level of TNF-α was found in the hydrogel composite group in comparison with control. On day 7, MMP-2 levels were higher in the hydrogel than control group.
[48]	CN	HEK, HUVEC cells, and type 1 diabetic mice were used.	In vitro: SEM images: Bacterial suspensions in the presence of HvCuO@GOx or PBS with different concentrations of glucose were performed separately at 37 °C under 180 rpm. Scratch assay and endothelial tubule formation: HEK cells were incubated in the presence of HvCuO@GOx, HvCuO, or PBS. All groups were treated with glucose for 2 h. In vivo: Diabetes was induced by an intraperitoneal injection of streptozotocin in mice. Staphylococcus aureus-infected wounds were divided into the following treatments: hydrogel, HSHvCuO, and HSHvCuO@GOx.	In vitro: SEM images, cytotoxicity assay, scratch assay, and endothelial tubule formation. In vivo: Wound healing and treatment, and imaging in vivo.	To design a thermal-responsive spray for the synergistic restoration of DFU using its angiogenesis and antibacterial properties.	Virus-like silica nanoparticles and Cu(NO_3_)_3_·6H_2_O were dissolved in deionized water and agitated for 30 min. Then, (CH_2_)_6_N_4_ was added, and the aforementioned mixture was constantly agitated for 2 h. Final samples were centrifuged multiple times with deionized water to eliminate unreacted residues, then dried in an oven. Finally, the virus-like mesoporous silica template was etched with 0.1 M Na_2_CO_3_, agitated, and washed three times with deionized water. HvCuO were produced after drying in an oven overnight.	In vitro: SEM images: In the NPs group, the rough surface was nicely maintained. No changes were found in the morphology of HvCuO@GOx nanoshells even without adding glucose for 12 h. After 2 h, the glucose concentration was dramatically reduced in the HvCuO@GOx presence in comparison with HvCuO. The most effective bactericidal action was found in HSHvCuO@GOx dressing. Cytotoxicity assay: A negligible cytotoxicity was found in HEK and HUVEC cells treated with different HvCuO@GOx concentrations. Scratch assay: A significant cell migration was found in HEK cells treated with HvCuO@GOx. Endothelial tubule formation: A higher number of tubule junctions was found in HUVEC cells treated with HvCuO@GOx at 150 μg/mL concentration. In vivo: Wound healing and treatment: At day 15, the wound healing was almost completely healed in the HSHvCuO@GOx group. Imaging in vivo: The highest quantity of CD34-positive cells was found in the HSHvCuO@GOx group.
[49]	CN	Recombinant human bFGF, NIH-313 cells, and HUVEC cells.	In vitro: Mechanical properties: A universal machine tester was used to evaluate the mechanical properties of the compounds. Antibacterial activity: The antibacterial ability of compounds was assessed using Escherichia coli and Staphylococcus aureus. Cell cytotoxicity and proliferation assessment and migration and tubule formation activities: Different ionic extractions were used to culture NIH/3T3 and HUVEC cells. In vivo: Rats were used to prepare the wound model. Each one was treated with GelMA, 5% Cu-NA@GelMA, or 5% Cu-NA-bFGF@GelMA. The skin was extracted for further histological and immunohistochemical studies on days 3, 7, and 14.	In vitro: Mechanical properties, antibacterial activity, cell cytotoxicity and proliferation assessment, and migration and tubule formation activities. In vivo: Wound healing and histopathologic evaluation.	To prepare a new Cu-nicotinic acid based on using biomolecules of nicotinic acid.	C_2_H_5_NO_2_ and Cu_2_(OAc)_4_(H_2_O)_2_ were mixed in deionized water and CH_2_OH solutions in various ratios, then heated and agitated. Cu_2_(OAc)_4_(H_2_O)_2_ solution was then swiftly added to the C_2_H_5_NO_2_ solution while rapidly swirling at 1000 rpm. After stirring, the solution was centrifuged, and then washed with deionized water and ethanol to get the copper-nicotinic acid. Finally, the blue powders were obtained by the freeze-drying technique and kept at 4 °C for future study.	In vitro: Mechanical properties: the best elasticity was found in the CuNA@GelMAs group. Antibacterial activity: CuNA@GelMAs showed good antibacterial ability toward E. coli and S. aureus. Moreover, an enhancement in antibacterial properties was observed after increasing the CuNA content in the hydrogels. Cell cytotoxicity and proliferation assessment: CuNA-bFGF@GelMA, CuNA@GelMA, and GelMA have shown no significant difference. Migration and tubule formation activities: In HUVEC and NIH/3T3 cells, increased migration and total segment length in the GelMA group were found. In vivo: Wound healing: In CuNA@GelMA and Cu-NA-bFGF@ GelMA treatments, a decreased percentage of wound closures was found in comparison to other groups. Histopathologic evaluation: More new blood vessels, regular epithelium, and mild inflammatory responses in CuNA@GelMA and CuNA-bFGF@GelMA treatments were found.
[50]	USA	Immortalized HEKas and HDF cells. Diabetic (db/db) mice were used.	In vitro: Cytotoxicity and apoptosis assays: Different concentrations of PPCN, H_3_BTC, CuSO4, H-CuSO4, HKUST-1 NPs, or H-HKUST-1 were used as treatments in HEKa and HDF cells. Cell migration assay: HEKa and HDF cells were used in a confluent monolayer. In vivo: Mice were separated into the following groups: PBS-treated, HKUST-1-treated, PPCN-treated, and H-HKUST-1-treated.	In vitro: Cytotoxicity, apoptosis, and scratch assays. In vivo: Wound healing and histopathological analysis.	To assess whether HKUST-1 NPs embedded within an antioxidant thermoresponsive citrate-based hydrogel would decrease Cu cytotoxicity and accelerate the healing process in a diabetic model.	As previously reported, the author’s group synthesized PPCN [51]. First, a PPCac was made by polycondensing C_2_H_8_O_7_, PEG, and C_9_H_12_O_5_. PPCac was then reacted with repurified NIPAM overnight by free radical polymerization using AIBN as the free radical initiator. Precipitation and purification with (C_2_H_5_)_2_O yielded the reaction product, PPCN. The PPCN was then dissolved in PBS, neutralized with NaOH to pH 7.4, and stored as a lyophilized powder for further use. HKUST-1 NPs were created using a previously described process (Xiao et al., 2013). To make a gel solution, Cu(CO_2_CH_3_)_2_ dissolved in distilled water was dropwise added to H3BTC diluted in ethanol, followed by stirring for 20 min. To get pure HKUST-1, the suspension was centrifuged, and the precipitate was washed with an ethanol/water solution. H-HKUST-1 was created by adding HKUST-1 NPs to a PPCN solution with Cu at room temperature.	In vitro: Cytotoxicity assay: A lower toxicity inH-HKUST-1 treatment was found (1 × 10^−3^ M). Apoptosis assay: Cell apoptosis was 10.7 ± 2.5% and 17.0 ± 5.4% in HEKa and HDF cells after H-HKUST-1 treatment. Cell migration assay: The highest cell migration after H-HKUST-1 treatment in HEKa and HDF cells was found. In vivo: Wound healing: On day 21, the wound almost completely healed after H-HKUST-1 treatment. However, PBS, PPCN, and HKUST-1 NP groups healed at days 39, 39, and 37, respectively. A faster healing time in H-HKUST-1 treatment in comparison to PBS, PPCN, and HKUST-1 NP groups was found. Histopathological analysis: A more stable and densely perfused vascular network in H-HKUST-1 and HKUST-1 NP treatment in comparison to PBS and PPCN groups was found. In the HKUST-1 and H-HKUST-1 groups, an increased blood vessel number and area, as well as neovascularization, were found. Higher granulation tissue and blood vessel numbers in HKUST-1- and H-HKUST-1-treated wounds were found, respectively. Smaller granulation tissue in the H-HKUST-1-treated wound was found.
[52]	USA	Immortalized HEKa, HDF, and HUVEC cells. Diabetic (db/db) and non-diabetic (C57BL/6) mice were used.	In vitro: Cytotoxicity and scratch assay: Folic acid, HKUST-1, and F-HKUST-1 were used as treatments in HEKa and HDF cells. Endothelial tubule formation assay: PBS, folic acid, HKUST-1, and F-HKUST-1 were used as treatments in HUVEC cells. In vivo: Three treatments (HKUST-1, F-HKUST-1, and folic acid) were used to evaluate wound healing at different time points.	In vitro: Cytotoxicity, scratch, and endothelial tubule formation assays. In vivo: Wound healing and histopathology analysis.	To evaluate the modification of HKUST-1, to release Cu^2+^, reducing cytotoxicity and improving wound healing rates.	HKUST-1 was synthesized in the manner previously reported (17, 46). Following the modification HKUST-1 synthesis technique to include C_19_H_19_N_7_O_6_ created F-HKUST-1. C_19_H_19_N_7_O_6_ in DMSO was combined with H_3_BTC in ethanol. The H_3_BTC/C_19_H_19_N_7_O_6_ solution was then treated with ethanol. To generate a green, gel-like suspension, Cu(CO_2_CH_3_)_2_ dissolved in deionized water was added dropwise to the H_3_BTC/C_19_H_19_N_7_O_6_ solution and agitated at room temperature. To get pure F-HKUST-1, the suspensions were centrifuged and the precipitates were washed with reaction media DMSO, ethanol, or water, respectively. Purified particles were kept at −80 °C in ethanol.	In vitro: Cytotoxicity assay: A lower toxicity in F-HKUST-1 treatment was found (0.5 mM). Scratch Assay: The highest migration rate in F-HKUST-1-treated cells was found. Endothelial tubule formation assay: The largest number of tubule junctions in F-HKUST-1-treated HUVEC cells was found. In vivo: Wound healing: At days 19, 21, and 30 post-wounding, an improved wound healing in the F-HKUST-1 group was found (*p* < 0.05). Histopathology analysis: At day 30, a 107.8 ± 18.1 μm granulation tissue thickness in the F-HKUST-1 group was found.
[53]	CN	HUVEC cells and female BALB/c mice were used.	In vitro: Antibacterial performance: For the optimal concentration, nanoliquid dressings at different concentrations were used (CuS nanoplates and HNO_3_). Anti-biofilm assay: Crystal violet assay was used. In vivo: Wound healing: Mice were separated into the following groups: control; HNO_3_; CuS; CuS + HNO_3_; CuS + NIR; and CuS + HNO_3_+NIR.	In vitro: Antibacterial performance and anti-biofilm assay. In vivo: Wound healing.	To design a novel nanoliquid dressing based on a mild photothermal heating strategy to provide safe healing of biofilm-infected wounds.	A container was filled with sulfur powder and 1-ODE. After the oxygen was removed, the mixture was heated. Then, the sulfur powder was dissolved and insulated for future use. In another container, there was CuCl_2_ powder, OM, and 1-ODE. When the CuCl_2_-containing mixture was heated in a vacuum, a brilliant yellow solution was produced. The container was then quickly injected into a sulfur-containing 1-ODE solution. In between injection cycles, CuS nanocrystals were cultured. After six injection sessions, CuS nanoplates were created. The generated CuS nanoplates were precipitated and centrifuged using an excess of ethanol after the reaction solution was cooled to room temperature. The precipitate was washed and kept at room temperature in chloroform for future use.	In vitro: Antibacterial performance: An ~80% cell viability in CuS-CTAB nanoplate-treated or HNO_3_-treated HUVEC cells was found. Anti-biofilm assay: A thickness reduction of biofilms in the CuS−HNO_3_ + NIR group was found in comparison to untreated biofilm. In vivo: Wound healing: At day 15, 72.9% and 98.6% of wound closure in normal saline and CuS−HNO_3_ + NIR-treated mice were found.

(C_2_H_5_)_2_O: ethyl ether; (CH_2_)_6_N_4_: hexamethylenetetramine; 1-ODE: octadecene; Ag NPs: silver nanoparticles; AuAg NSs: gold−silver core nanoshell; AuAg: gold−silver core; AuAgCu_2_O NSs: hollow gold−silver core and cupric oxide nanoshell; AuAgCu_2_O: hollow gold−silver core and cupric oxide; BACA/Cu NPs: N, N-bis(acryloyl) cystamine-chelated copper nanoparticles; BC: bacterial cellulose; bFGF: basic fibroblast growth factor; BSA: bovine serum albumin; C_13_H_21_NO_15_S: Chondroitin sulfate; C_14_H_21_NO_11_)n: hyaluronic acid; C_19_H_19_N_7_O_6_: folic acid; C_30_H_48_O_5_: asiatic acid; C_2_H_5_NO_2_: nicotinic acid; C_2_H_8_O_7_: citric acid; C_9_H_12_O_5_: 5-Methyl-2-oxo-1,3-dioxane-5-carboxylic acid; Ca(NO_3_)_2_·4H_2_O: calcium nitrate tetrahydrate; CAM: chick chorioallantoic membrane; CH_2_OH: methyl alcohol; Cu NPs: copper nanoparticles; Cu-MBG: copper mesoporous bioactive glasses; Cu: copper; Cu(CO_2_CH_3_)_2_: cupric acetate; Cu(NO_3_)_2_: copper(II) nitrate; Cu(NO_3_)_3_·6H_2_O: cupric nitrate hexahydrate; Cu_2_(OAc)_4_(H_2_O)_2_: copper(II) acetate; Cu^2+^: copper; Cu_5.4_O USNPs: ultrasmall cupric oxide nanoparticles; CuCl_2_: cupric chloride; CuNA-bFGF@GelMA: gelatin methacrylate loaded with copper-nicotinic acid+ basic fibroblast growth factor; CuNA@GelMA: gelatin methacrylate loaded with copper-nicotinic acid; CuNA: copper-nicotinic acid; CuO: cupric oxide; CuS NDs: copper sulfide nanodots; CuS-CTAB: copper sulfide dressings loaded with hexadecyltrimethylammonium bromide; CuS: copper sulfide; CuS + HNO_3_: copper sulfide dressings loaded with nitric acid; CuS + HNO_3_ + NIR: copper sulfide dressings loaded with nitric acid and exposed to near-infrared laser light; CuS + NIR: copper sulfide dressings exposed to near-infrared laser light; CuSO_4_·_5_H_2_O: copper sulfate pentahydrate; CuSO_4_: copper sulfate; DFU: diabetic foot ulcer; DMEM: Dulbecco’s minimal essential medium; DMSO: dimethyl sulfoxide; EDC: 1-Ethyl-3-(3-dimethylaminopropyl)carbodiimide; F-HKUST-1: folic acid-modified copper-based metal–organic framework; FTIR: Fourier transform infrared; GAGs: glycosaminoglycans; GelMA: gelatin methacrylate; GelMA/BACA-Cu NPs: N, N-bis(acryloyl) cystamine-chelated copper nanoparticles loaded with gelatin methacrylate; H-CuSO_4_: copper sulfate ion-containing hydrogel; H-HKUST-1: copper-based metal–organic framework-hydrogel; H_3_BTC: benzene-1,3,5-tricarboxylic acid; HaCaT: human keratinocytes; HCEPCs: human corneal epithelial cells; HDFs: human dermal fibroblasts; HEKs: human epidermal keratinocytes; HEK293: human embryonic kidney 293; HEKas: human epithelial keratinocytes; HFF-1: human foreskin fibroblast cell; HKUST-1 NPs: copper-based metal–organic framework nanoparticles; HKUST-1: copper-based metal–organic framework; HNO_3_: nitric acid; HUVECs: human umbilical vein endothelial cells; HvCuO: hollow virus-like mesoporous cupric oxide; HvCuO@GOx: glucose oxidase loaded with hollow virus-like mesoporous cupric oxide; Hydrogel + CuNPs: hydrogel with copper nanoparticles; Hydrogel + CuNPs + NIR: hydrogel with copper nanoparticles exposed to infrared laser light; L929: fibroblast cell line; MBG: mesoporous bioactive glasses; MMP-2: matrix metalloproteinase-2; MTT: 3-[4,5-dimethylthiazol-2-yl]-2,5 diphenyl tetrazolium bromide; N_2_H_4_·3H_2_O: hydrazine trihydrate; NA: nicotinic acid; Na_2_CO_3_: sodium carbonate; Na_3_C_6_H_5_O_7_: trisodium citrate; NaBH4: sodium borohydride; NaOH: sodium hydroxid; NDs: nanodots; NH_3_: ammonia; NH_4_OH: ammonium hydroxide: NIR: infrared laser light; NPs: nanoparticles; OM: oleylamine; PBS: phosphate-buffered saline; PEG: polyethylene glycol; PPCac: poly (polyethyleneglycol citrate) acrylate prepolyme; PPCN: poly(polyethyleneglycol citrate-co-N-isopropylacrylamide); PTT: photothermal therapy; PVP: polyvinylpyrrolidone; RBC: red blood cells; ROS: reactive oxygen species; SEM: scanning electron microscopy; USNP: ultrasmall nanoparticles; XRD: X-ray diffraction; ZnO: zinc oxide.

**Table 2:** Variables involved in effectiveness of copper nanoparticles in the healing process of chronic wounds in in vivo and in vitro studies.

Measurement	Wound Changes	Evaluation Method	References
Cytotoxicity	Cytotoxicity of Cu-MBG was well-tolerated (at 0.1 and 1 mg/mL).	Metabolic assay PrestoBlue^TM^	[42]
	The generation of hydroxyl radicals in the biofilm environment may be of insignificant toxicity in diabetic wounds.	Endothelial tubule formation	[48]
	Good in vivo safety and biocompatibility have been suggested after CuS NDs + NIR treatment, where no histological changes or toxicity within the treatment period were found.	H&E staining	[43]
	After treatment with HKUST-1 and F-HKUST-1, HEKas and HDF cells exhibited enhanced migration. In addition, the highest cell migration in F-HKUST-1 has been found. Its enhanced migration is due to the Cu^2+^ presence in HKUST-1 and F-HKUST-1 groups.	Scratch assay	[52]
	About NIH-3T3 cells, no significant in vitro cytotoxicity has been described.	CCK-8 assay	[45]
	On day 28, no differences in the inflammatory cells were found.	Haematological analysis	[46]
	The use of hydrogel + NPs treatment in second-degree burns is safe because no changes in markers of liver and kidney have been found.	Biochemistry analysis	[46]
	No significant cytotoxicity after treatment with different concentration of USNPs was found. Good biocompatibility and normal cytoskeleton morphology in HEK293 cells treated with USNPs (200 ng mL^−1^) have been described.	CCK-8 assay	[41]
	After 30 days, no cardiovascular damage after USNP injection has been found.	Hemolysis assay	[41]
	After 24 h, no tissue damage or inflammatory lesions in the genitourinary system after USNPs injection have been described.	Hemolysis assay	[41]
	A significant inhibition in the MAPK pathway after USNPs treatment was found, showing it might decrease renal injury by decreasing the ROS level.	Principal component analysis	[41]
	As the incubation period wore on, the number of cells grew for all groups. The viability of cells decreased for the BC/Cu_60_- and BC/Cu_100_-treated groups (46 ± 6% and 30 ± 8%, respectively). After BC/Cu_20_ treatments, NHDF cells did not show decreased cell viability in comparison to the control group; after BC/Cu_60_ and BC/Cu_100_ treatments, NHDF cells showed decreased cell viability.	CCK-8 assay and calcein staining	[40]
Antibacterial response	Antibacterial effects of Cu-MBG (100 μg/mL) against *Pseudomonas aeruginosa* and *Staphylococcus aureus* with 1.2–3.5 log reductions were found. In addition, a reduced existing biofilm after Cu-MBG treatment was described.	Brain heart infusion	[42]
	Eradication of bacteria after CuS NDs (45 μg/mL) treatment was found. Likewise, a higher antibacterial effect in the CuS NDs+NIR group than in the CuS NP + NIR group was found.	Growth-inhibition assay	[43]
	In bacteria after CuS NPs+NIR and CuS NDs treatments, outer membranes were damaged. However, in bacterial cell walls, a loss of integrity after CuS NDs+NIR treatment was found. In addition, after CuS NDs+NIR treatment, the cytoplasm displayed aggregates in *Escherichia coli* and *Staphylococcus aureus*, confirming the cell damage.	TEM	[43]
	After incubation with AuAgCu_2_O NSs, several dead cells were detected in the *Escherichia coli* incubation; however, almost complete death of bacteria after AuAgCu_2_O NSs treatment with a laser was found.	SYTO9/PI live/dead fluorescent staining assay	[44]
	After 6 and 24 h of incubation, effective antibacterial effects in GelMA/BACA-CuNPs hydrogels+NIR against *Escherichia coli* and *Staphylococcus aureus* have been described.	In vitro antibacterial assay	[45]
	The inhibition zone for *Escherichia coli* and *Staphylococcus aureus* was 3.1 ± 0.8 mm and 2.6 ± 0.3 mm for the hydrogel; it was 5.3 ± 0.2 mm and 4.9 ± 0.6 mm for the gelatin + ZnO group, whereas the inhibition zone was 4.8 ± 0.7 mm and 3.8 ± 0.3 mm for the gelatin + CuO treatment.	Disc diffusion method	[46]
	BC/Cu membranes showed a higher inhibition zone against *Staphylococcus aureus* for up to 90 days.	Disk diffusion method	[40]
	A slow release of Cu in membranes was found even at 90 days, and remaining membranes with Cu were found. However, a faster release of Cu was found when a 37 °C incubator was used; after 24 h, the release of Cu of membranes was almost complete.	Disk diffusion method	[40]
Wound healing	At 24 h, the outgrowth of endothelial cells started for most aortic rings, and the outgrowth area increased from 0.3 to 1.5 mm^2^ when VEGF was added.	Aortic ring assay	[42]
	Increases in the number of junctions and total vessel length in MBG and Cu-MBG groups were found.	CAM assay	[42]
	The vascular network formation was promoted by H-HKUST-1 and HKUST-1 NP treatments. In addition, blood vessel area, blood vessel number, and neovascularization in HKUST-1-and H-HKUST-1 were increased.	OCTA	[50]
	The highest functional levels of blood vessel oxygen of the HSHvCuO@GOx group were found, confirming the properties of HSHvCuO@GOx in hypoxia alleviation.	Photoacoustic imaging in vivo	[48]
	A total of 14 days was necessary for the F-HKUST-1 group to close 50% of the wound area, whereas a total of 19 days was necessary for the other groups.	Dermal excision wound model	[52]
	Tight connections, parallel cell lines, and mesh circles in AuAgCu_2_O NSs treatment groups with or without laser irradiation indicated a late phase of angiogenesis.	Matrigel assay	[44]
	Effective antibacterial capacity in hydrogel + CuNPs + NIR after CD86+ and CD206 intensity analysis was found.	IHC	[45]
	A larger degradation rate in Cu NP hydrogels than in simple hydrogels was found.	Masson’s trichrome and H&E staining	[45]
	On day 7, decreased inflammation and high tissue remodeling as a consequence of low levels of TNF-α and high levels of MMP-2 in the hydrogel composite were found.	ELISA kit	[46]
	A faster healing rate in the USNPs group in comparison to the control group was found during days 4, 7, 9, and 15 post-surgery (*p* < 0.01).	H&E staining	[41]

BC/Cu: bacterial cellulose; BC/Cu_20_: 20 mM cupric chloride nanoparticles loaded with bacterial cellulose; BC/Cu_60_: 60 mM cupric chloride nanoparticles loaded with bacterial cellulose; BC/Cu_100_: 100 mM cupric chloride nanoparticles loaded with bacterial cellulose; CAM: chick chorioallantoic membrane; CuNPs: copper nanoparticles; Cu-MBG: copper mesoporous bioactive glasses; Cu^2+^: copper; Cu_5.4_O; USNPs: ultrasmall cupric oxide nanoparticles; CuO: cupric oxide; CuS NDs: copper sulfide nanodots; CuS NPs: copper sulfide nanoparticles; ESBL: extended-spectrum *β-lactamase*; F-HKUST-1: folic acid-modified copper-based metal–organic framework; GelMA/BACA-Cu NPs: N, N-bis(acryloyl) cystamine-chelated copper nanoparticles loaded with gelatin methacrylate; H-HKUST-1: copper-based metal–organic framework hydrogel; H&E: Hematoxylin and Eosin; HDFs: human dermal fibroblasts; HEK293: human embryonic kidney 293; HEKas: human epithelial keratinocytes; HKUST-1: copper-based metal–organic framework; HKUST-1 NPs: copper-based metal–organic framework nanoparticles; HSHvCuO: hydrogel spray after the incorporation of hollow virus-like mesoporous cupric oxide; HSHvCuO@GOx: hydrogel spray after the incorporation of glucose oxidase loaded with hollow virus-like mesoporous cupric oxide; IHC: immunohistochemistry; MAPK: mitogen-activated protein kinase; MBG: mesoporous bioactive glasses; MMP-2: matrix metalloproteinase-2; NHDFs: normal human dermal fibroblasts; NIR: near-infrared laser light; OCTA: optical coherence tomograph angiography; PBS: phosphate-buffered saline; PPCN: poly(polyethyleneglycol citrate-co-N-isopropylacrylamide); PI: propidium iodide; TNF-α: tumor necrosis factor alpha; VEGF: vascular endothelial growth factor; ZnO: zinc oxide.

**Table 3:** National Institute for Health and Care Excellence Methodology Checklist: quantitative studies.

References	Study Design	Population			Method of Allocation to Intervention (or Comparison)										Outcomes						Analyses						Summary	
		1	2	3	4	5	6	7	8	9	10	11	12	13	14	15	16	17	18	19	20	21	22	23	24	25	26	27
[40]	Clinical trial	++	++	+	++	++	NR	NA	++	++	+	++	NA	NA	++	++	++	++	+	++	++	NR	+	++	++	+	++	+
[41]	Clinical trial	++	+	+	+	++	NR	NR	+	+	NR	++	NA	NA	++	++	++	++	++	++	++	NR	+	++	++	++	++	+
[42]	Clinical trial	++	+	+	NR	++	NR	NR	+	+	NA	NA	NA	NA	++	++	++	++	++	++	NR	NR	+	+	++	++	++	++
[43]	Clinical trial	++	+	+	NR	++	NR	NR	++	++	++	++	NA	NA	++	++	++	++	++	++	++	NR	++	++	++	++	++	++
[44]	Clinical trial	++	+	+	NR	++	NR	NR	++	++	++	++	NA	NA	++	++	++	++	++	++	+	NR	+	++	++	++	++	++
[45]	Clinical trial	++	+	+	NR	++	NR	NR	++	++	++	+	NA	NA	++	++	++	++	++	++	++	NR	++	++	++	++	++	++
[46]	Experimental study	++	+	+	++	++	NR	NA	++	++	+	++	NA	NA	++	++	++	++	++	++	++	NR	++	++	++	++	++	++
[48]	Clinical trial	++	+	+	NR	++	NR	NR	++	++	NR	++	NA	NA	++	++	++	++	++	++	NR	NR	++	+	++	++	++	++
[49]	Experimental study	+	+	+	+	++	NR	NA	++	+	+	++	NA	NA	++	++	++	++	++	++	NR	NR	++	+	++	++	++	++
[50]	Clinical trial	++	+	+	++	++	NR	NR	++	++	++	++	NA	NA	++	++	++	++	++	++	++	NR	+	+	++	++	++	++
[52]	Clinical trial	++	+	+	NR	+	NR	NR	++	++	+	++	NA	NA	++	++	++	++	++	++	+	NR	+	++	++	++	++	++
[53]	Clinical trial	++	+	+	++	++	NR	NA	++	++	++	++	NA	NA	++	++	++	++	++	++	+	NR	++	+	++	+	++	++

**Key to headings: Population** 1. Is the source population or source area well described? 2. Is the eligible population or area representative of the source population or area? 3. Do the selected participants or areas represent the eligible population or area? **Method of allocation to intervention (or comparison)** 4. Allocation to intervention (or comparison). How was selection bias minimized? 5. Were interventions (and comparisons) well-described and appropriate? 6. Was the allocation concealed? 7. Were participants or investigators blind to exposure and comparison? 8. Was the exposure to the intervention and comparison adequate? 9. Was contamination acceptable now? 10. Were other interventions similar in both groups? 11. Were all participants accounted for at study conclusion? 12. Did the setting reflect usual UK practice? 13. Did the intervention or control comparison reflect usual UK practices? **Outcomes** 14. Were outcome measure reliable? 15. Were all outcome measurements complete? 16. Were all important outcomes assessed? 17. Were outcomes relevant? 18. Were there similar follow-up times in exposure and comparison groups? 19. Was follow-up time meaningful? **Analyses** 20. Were exposure and comparison groups similar at baseline? If not, were these adjusted? 21. Was intention to treat (ITT) analysis conducted? 22. Was the study sufficiently powered to detect an intervention effect (if one exists)? 23. Were the estimates of effect size given or calculable? 24. Were the analytical methods appropriate? 25. Was the precision of intervention effects given or calculable? Were they meaningful? **Summary** 26. Are the study results internally valid (i.e., unbiased)? 27. Are the findings generalizable to the source population (i.e., externally valid)? (National Institute for Health and Care Excellence (NICE) Methodology checklist: quantitative studies. https://www.nice.org.uk/process/pmg4/chapter/appendix-f-quality-appraisal-checklist-quantitative-intervention-studies, accessed on 1 April 2022). Not applicable (NA): It is reserved for those study design aspects that are not applicable given the study design under review; not reported (NR): it is reserved for those aspects in which the study under review fails to report how they have (or might have) been considered; -: it is reserved for those aspects of the study design in which significant sources of bias may persist; +: it indicates that either the answer to the checklist question is not clear from the way the study is reported, or that the study may not have addressed all potential sources of bias for that particular aspect of study design; ++: it indicates that for that particular aspect of study design, the study has been designed or conducted in such a way as to minimize the risk of bias.

## Data Availability

Not applicable.
